# How Best to Obtain Valid, Verifiable Data Online From Male Couples? Lessons Learned From an eHealth HIV Prevention Intervention for HIV-Negative Male Couples

**DOI:** 10.2196/publichealth.6392

**Published:** 2016-09-12

**Authors:** Jason Mitchell, Ji-Young Lee, Rob Stephenson

**Affiliations:** ^1^ Office of Public Health Studies University of Hawai'i at Manoa Honolulu, HI United States; ^2^ Center for Sexuality and Health Disparities Department of Health Behavior and Biological Sciences University of Michigan School of Nursing Ann Arbor, MI United States

**Keywords:** eHealth, male couples, dyadic data, recruitment, validation, verification

## Abstract

**Background:**

As interest increases in the development of eHealth human immunodeficiency virus (HIV)-preventive interventions for gay male couples, Web-based methods must also be developed to help increase the likelihood that couples enrolled and data collected from them represent true unique dyads. Methods to recruit and collect reliable and valid data from both members of a couple are lacking, yet are crucial for uptake of novel sexual health and HIV-prevention eHealth interventions. Methods to describe best practices to recruit male couples using targeted advertisements on Facebook are also lacking in the literature, yet could also help in this uptake.

**Objective:**

The objective of our study was to describe challenges and lessons learned from experiences from two phases (developmental phase and online randomized controlled trial [RCT]) of an eHealth HIV-prevention intervention for concordant HIV-negative male couples in terms of (1) recruiting male couples using targeted advertisements on Facebook, (2) validating that data came from two partners of the couple, and (3) verifying that the two partners of the couple are in a relationship with each other.

**Methods:**

The developmental phase refined the intervention via in-person focus groups, whereas the pilot-testing phase included an online RCT. For both phases, couples were recruited via targeted Facebook advertisements. Advertisements directed men to a study webpage and screener; once eligible, participants provided consent electronically. A partner referral system was embedded in the consenting process to recruit the relationship partner of the participant. Both men of the couple had to meet all eligibility criteria—individually and as a couple—before they could enroll in the study. Verification of couples’ relationships was assessed via the concurrence of predetermined screener items from both partners, done manually in the developmental phase and electronically in the pilot-testing phase. A system of decision rules was developed to assess the validity that data came from two unique partners of a couple.

**Results:**

Several important lessons were learned from these experiences, resulting in recommendations for future eHealth studies involving male couples. Use of certain “interests” and types of images (eg, shirtless) in targeted Facebook advertisements should be avoided or used sparingly because these interests and types of images may generate adverse reactions from a broader audience. Development of a systematic approach with predetermined criteria and parameters to verify male couples’ relationships is strongly recommended. Further, researchers are encouraged to develop a system of decision rules to detect and handle suspicious data (eg, suspicious email addresses/names, multiple entries, same IP address used in multiple entries) to help validate the legitimacy of male couples’ relationships online.

**Conclusions:**

These lessons learned combined with recommendations for future studies aim to help enhance recruitment efforts and the validity and reliability of collecting dyadic data from male couples for novel eHealth HIV-preventive interventions.

## Introduction

Use of the Internet, including social media, has dramatically increased within the past decade. As evidenced in the Pew Research Center’s Internet and American Life Project, the prevalence of Internet use among US adults rose from 14% to 87% between 1995 and 2014 [1], and social media use increased from 8% to 76% between 2005 and 2015 [2]. Not only has the Internet allowed individuals and communities to connect and communicate via various multimedia features (eg, chatting, picture sharing, messaging), it has also increasingly been used as a platform to disseminate health information and promote the uptake of healthy behaviors across all scientific disciplines [3-5]. These online environments have created a wide yet complex venue to recruit and collect psychosocial and behavioral data to further health promotion and prevention efforts across different populations.

For example, the Internet has increasingly been used for human immunodeficiency virus (HIV) prevention research with various populations due to the efficiency of targeting and collecting data from specific subpopulations, such as gay, bisexual, and other men who have sex with men (MSM) [6-10]. Despite this efficiency, characteristics of MSM who are recruited online versus in person may differ for some characteristics and not in others. For example, MSM recruited online versus venue-based, time-spaced sampling (ie, offline) did not statistically differ regarding their HIV and sexually transmitted infection (STI) testing patterns, prevalence of HIV and other STIs, and study retention rates up to 24 months [11]. However, online studies with MSM and male couples suggest that Internet-based samples of MSM tend to self-report as being more educated and non-Hispanic white [12,13]. As such, online studies with MSM and male couples may limit the potential for generalizability depending on who participates in the research study.

With respect to prevention, some MSM use the Internet to meet other MSM for sex, friendships, relationships, and for various types of social support [3,14,15]. Although MSM are not the only population to use the Internet for these reasons, research has also noted that Internet use has been associated with having higher numbers of sex partners, greater frequency of condomless anal sex (receptive and insertive), and increased levels of recreational drug use (eg, methamphetamines) among MSM [16,17]. Given these reasons, HIV-prevention efforts that are conducted online may be beneficial to reach some of these MSM in addition to the efforts that are provided in person.

Although methods of recruiting online are more time efficient than recruiting in person, there are challenges related to the reliability and validity of collecting data online from participants [18]. For example, anonymity and lack of direct face-to-face contact with participants prohibit researchers to know who and where data are originating from when it is collected online. In addition, randomly generated responses to survey items may occur in order to help expedite completion of the survey, particularly if an incentive is involved. Monetary incentives have been frequently associated with increased participant misrepresentation for eligibility [19,20] and multiple data entry [20,21] by individuals who wish to increase the amount of incentives and/or probabilities of winning. Survey response rates may also decrease over time, which could threaten the validity of generalizing the results to a broader audience. Bauermeister and colleagues [22] examined how invalid data collected online may influence statistical relationships, decrease statistical power, and increase the likelihood of biased conclusions. They did not recommend using a conservative approach and excluding suspicious entries because valid data could accidentally be removed in the process. Instead, they recommended developing both pre and post hoc decisions to handle all data, such as grouping entries into different categories (eg, valid, suspicious, and invalid) and obtaining a population list of all eligible participants for verification processes, respectively [22].

These issues of reliability and validity of online data become more complex when conducting online research with dyads (eg, gay male couples). By nature, handling dyadic data requires at least double the amount of time and effort than that required of individual-level data. The criteria used to determine the reliability and validity of an individual participant must be assessed not only twice, but must also be crosschecked with that of that participants’ partner’s data to (1) validate the data are coming from two unique individuals and (2) verify that these two unique individuals are in a relationship together. The eligibility criteria used in couples Web-based research is also inherently more complex due to the need to ensure that both individual- and couple-level eligibility requirements are met. However, the anonymity that the Internet warrants makes it easier for participants to pose as a fraudulent “couple” by pretending to be both partners in the relationship. If incentives are used, then this further increases the need to ensure that dyadic data collected online are valid and reliable because some couples’ motivation to become eligible may be less than honest. For these reasons and due to the increased use of the Internet among adults, methods are needed to help ensure that dyadic data collected online from couples are valid and verifiable of their relationships.

In recent years, attention toward male couples for HIV prevention has increased because many MSM in the United States—up to 67%—acquire HIV while in a same-sex relationship [23,24]. In addition, few preventive interventions approved by the Centers for Disease Control and Prevention currently exist for this population [25-28]. Despite how common Internet use is among MSM and its efficiency to enhance recruitment and data collection efforts for prevention research, few studies have fully harnessed the capabilities of eHealth (ie, Web-based research and/or preventive programs hosted online) to help advance HIV-prevention efforts for male couples [29]. Moreover, the majority of HIV-preventive interventions for MSM have focused on behavior change at the individual level [30-33], thereby emphasizing the need to not only develop eHealth HIV-preventive interventions for male couples, but to also develop methods to maximize recruitment efforts and verification of valid data from both members of the couple (ie, dyadic data) online. Methods to recruit and collect reliable and valid dyadic data online are lacking, yet are crucial for development and uptake of future eHealth HIV-preventive interventions for male couples.

To help fill this critical gap, this paper aims to provide methodological recommendations in three distinct areas to (1) maximize efficiency and accuracy of using targeted, online Facebook advertisements to recruit male couples; (2) facilitate the collection of *valid* dyadic data; and (3) ensure verification that dyadic data collected are representative of two males who are in a relationship together (ie, male couple). To accomplish these aims, data and related experiences captured from the development and pilot testing of an eHealth HIV-prevention intervention with concordant HIV-negative male couples in the United States are used. The methods used and lessons learned from these experiences provide concrete suggestions and recommended safeguards that may benefit future eHealth endeavors targeting similar populations of male couples.

## Methods

### Procedure Overview

The University of Miami Institutional Review Board approved all study procedures. For both the development and pilot testing of the eHealth HIV-prevention intervention, interested men who clicked on the Facebook advertisement were directed to the study webpage and an eligibility screener via SurveyGizmo, a Health Insurance Portability and Accountability Act-compliant Web-based survey tool and database server. Once eligible, participants provided consent electronically. A partner referral system was embedded in the consent process to recruit the relationship partner of the index participant. Both men of the couple had to meet all eligibility criteria and be deemed valid—as an individual and as a couple—before they could enroll into either the development or pilot-testing portion of the study; the development portion included both partners of the couple participating in one in-person focus group, whereas the pilot testing of the intervention included a Web-based randomized controlled trial (RCT) with couples participating online.

### Targeted Recruitment via Facebook

Male couples were recruited via targeted advertisements placed on Facebook. All advertisements targeted potential research participants who were male, living in the United States, at least 18 years of age, interested in men, and had a relationship status of either being married, engaged, in a relationship, domestic partnership, or civil union. Each advertisement included a picture of a male couple with a brief title, message, and a Web link to the study eligibility screener. In total, 13 advertisement campaigns were conducted for the focus group, development phase, and RCT pilot-testing phase of the project, and each Facebook advertisement campaign lasted for 72 hours. The campaigns used to recruit for the focus groups occurred between March 2015 and May 2015, whereas the campaigns used to recruit for the online RCT occurred between October 2015 and March 2016. Following Facebook’s guidelines and word limits, all advertisements contained titles similar to “In a relationship?” with a brief message such as “Male couples wanted to try out a cool, new online health & relationship program. Earn $$!” Each campaign’s total cost ranged from US $499.97 to US $3997.62 depending on performance, audience, and placement of the advertisements (eg, desktop newsfeed, mobile newsfeed). Per campaign, the advertisements resulted in a mean of 110,478 people reached and a mean of 3534 clicks to the study website. Each targeted Facebook advertisement campaign provided useful metrics about how well the targeted advertisement performed, including (1) the total number of people reached, (2) number of people who clicked on the advertisement, (3) how relevant the targeted audience thought the advertisement pertained to them (ie, relevancy score ranging from 0 to 10 with 10 being most relevant), and (4) negative and positive feedback about the advertisement obtained from those who were shown the advertisement while using Facebook.

### Eligibility Criteria

Both members of the male couple had to meet the following eligibility criteria to participate in the focus group or RCT of the intervention project: (1) self-report as male, (2) be at least 18 years of age, (3) be in a current sexual relationship with a main relationship partner for at least 6 months, (4) self-report as HIV-negative, (5) practice condomless anal sex with the main relationship partner for at least 6 months, (6) self-report no recent history of intimate partner violence or coercion within the previous year, (7) have not established a sexual agreement in the relationship, and (8) own a mobile phone and have an alternate method to access the Internet (eg, computer).

### Procedures Used to Verify Couples’ Relationships and Validity of Dyadic Data Collected

**Figure 1:**
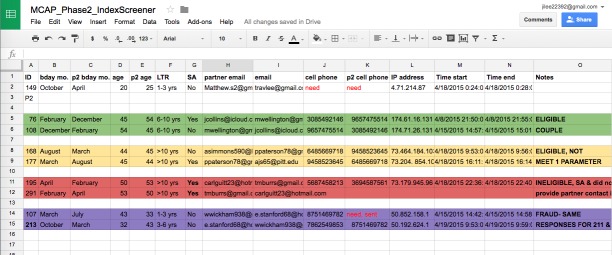
An example of some of the items used in the verification of couples’ relationships via manual analysis of the eligibility data collected from both partners online. Predetermined variables with decision rules were used to verify whether two partners were in a relationship with each other. Note: data represented in this figure are fictitious.

Both individual- and couple-level criteria were used to deem couples’ eligibility to participate in the study. Based on our prior work with male couples [34,35], we developed a system of decision rules to assess the verification of a couples’ relationship based on both partners’ responses to screener items (ie, to verify that a couple is in a relationship). Verification of couples’ relationships was based on data collected from both members of the couple and whether they concurred (ie, agreed) on certain predetermined eligibility screener items (see Figure 1), including (1) matching their relationship length within a margin of 1 month, (2) exact matching of each other’s birthday months, (3) matching each other’s ages within a margin of one year, and (4) exact matching of at least one of their two contact information options (eg, phone number, email address). All couples had to concur on all four items to be considered eligible. Each participant and his partner’s data were crosschecked to match and concur on all four of the predetermined screener items. Suspicious cases or cases in which couples did not concur on one or two items were assessed a second time after calling or emailing the potential participant to verify their corresponding partners’ information. Verification of couples’ relationships was done manually by entering data into a secure database during the developmental focus group phase; for the pilot RCT, this verification step was done electronically via an embedded Web-based system that contained an algorithm.

For both phases of the intervention project, validation of both partners’ data collected was assessed on a case-by-case basis. For the development phase, multiple entries of the same Internet Protocol (IP) address were examined to validate whether or not one or both partners were unique individuals. For example, each entry of the eligibility screener had an IP address associated with it. Some IP addresses were associated with several entries, potentially suggesting that individuals had attempted to complete the eligibility screener multiple times as either the same person or pretending to be different people. Eligibility screener entries with the same IP address were flagged and noted for further investigation by using the other validation markers.

Because the online pilot RCT aimed to recruit a large number of male couples (compared to the focus groups), an electronic algorithm was created to electronically match both partners as a couple based on predetermined eligibility criteria and verification rules. For the online RCT phase, suspicious names and/or email addresses, duplicate entries (ie, same two email addresses used for two different sets of couples), and similar back-to-back screener entries were examined in addition to the validation markers used in the developmental focus group phase.

## Results

Lessons learned from this intervention project are an example of the challenges associated with collecting and verifying valid dyadic data from male couples online. The lessons learned from this project are not exhaustive, yet provide insights and suggestions to help improve these efforts, particularly with the increased attention toward developing eHealth and mHealth preventive interventions for male couples [36-38]. Specifically, lessons learned from this project entail (1) advantages and disadvantages of using certain selection criteria to target advertisements on Facebook, (2) selection of images that resonate with the targeted audience, (3) use of an algorithm versus manual input of data to verify male couples’ relationships via predetermined decision rules, and (4) monitoring of dyadic data collected to assess validity of unique responses obtained from both partners of the male couple.

### Targeted Advertisements for Male Couples on Facebook

**Figure 2:**
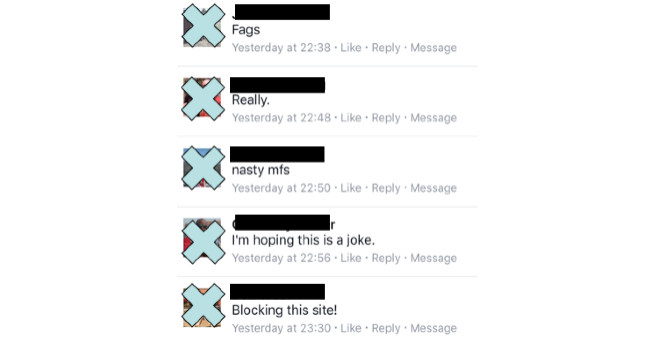
Examples of negative feedback received for some of the targeted advertisements used on Facebook.

Some images used in the advertisements received higher relevancy scores than others and received less negative feedback from supportive individuals who were not lesbian, gay, bisexual, and transgender (LGBT). Negative feedback was received in three forms: (1) public comments posted on the Facebook advertisements and study Facebook community page, (2) messages sent privately to the study Facebook community page, and (3) voicemail. Figure 2 provides examples of the negative feedback received from some of the images used for the targeted advertisements placed on Facebook.

**Figure 3:**
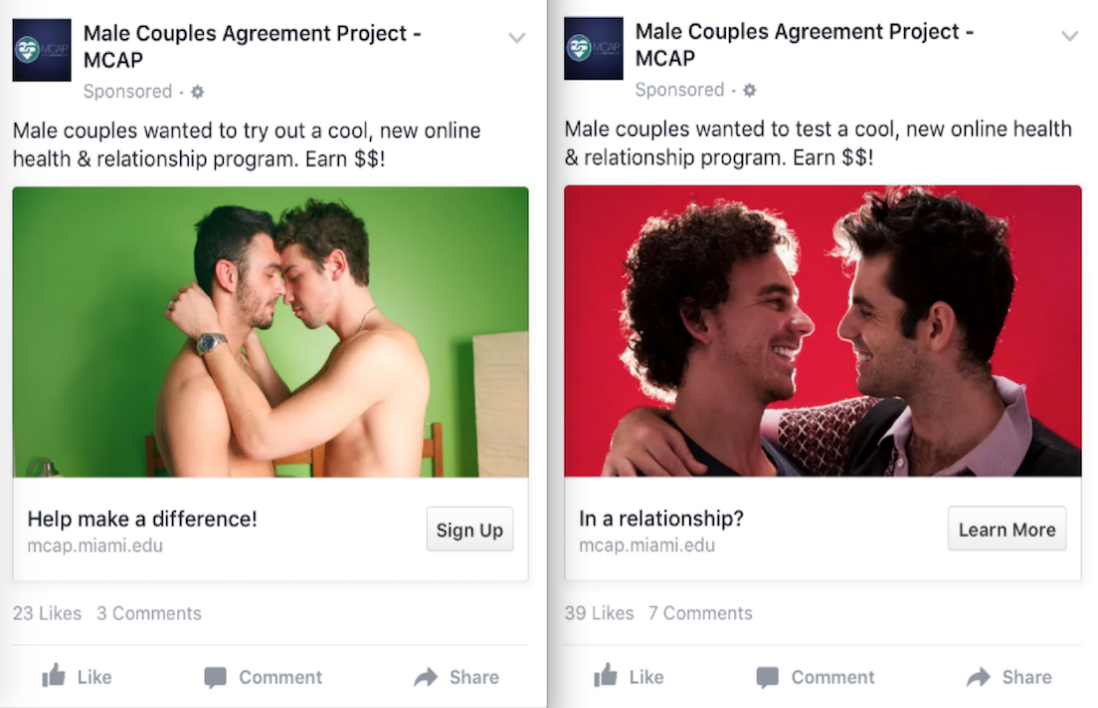
Example of Facebook advertisements before and after modifications. Image on the left was used before negative feedback was received. Image on the right was used after modifications were made to the images and the targeting criteria.

After some investigation and consultation with Facebook (private telephone communication with Facebook, January 13, 2016), interests that were used to target Facebook members, such as the LGBT community or gay news, included members who had positive views of this interest as well as those who had negative views of this same interest. Because the negative-view individuals were also targeted, this resulted in a flood of backlash in the form of unsupportive and homophobic comments posted on the study Facebook webpage, study team’s voicemail, and as comments left in association with the targeted advertisement. After these experiences, targeting criteria were adjusted for the remainder of the advertisement campaigns by actively excluding Facebook members who had the following information on or associated with their profiles: interested in women or both men and women; had a relationship status as single, unspecified, open, complicated, separated, divorced, or widowed; and had moderate, conservative, and very conservative political views. Targeted advertisements were also changed by using less overtly sexual images of male couples (eg, clothed male couple slightly embracing). Figure 3 provides examples of Facebook advertisements that were used before and after these modifications were made.

### Verification of Couples’ Relationships

A systematic manual (ie, color-coded) approach was used during the project’s developmental phase to verify whether both partners of the male couple were in a relationship (after both men had electronically consented) using predetermined eligibility screener items. This post hoc comparative method of the dyadic data collected from the eligibility screener enabled the characterization of couples into one of three groups: (1) eligible and verified couples who met all predetermined decision rules, (2) eligible but unverified couples because only two or three of the predetermined decision rules were met, and (3) ineligible couples. This systematic manual method illuminated the strengths and limitations of this approach to verify couples’ relationships. Although manually matching entries allowed for greater control over the assessment of couples’ relationships, this method proved inefficient in terms of time and resources.

Based on this experience, the amount of time required to verify couples’ relationships with this approach emphasized the necessity to electronically automate this process, particularly if data were to be collected online from large samples of male couples such as during an eHealth intervention. As such, a Web-based electronic algorithm was created to automatically verify couples’ relationships based on data collected from both members of the couple and whether they concurred on predetermined eligibility screener items. The algorithm was developed based on the results and experiences obtained during the developmental focus group phase. However, the algorithm had an important limitation. The system would report couples as partially matched when their contact information mismatched (eg, one partner reported his partners’ email address different from what his partner reported as his actual email address). This mismatch required a manual verification of the couples’ relationship by contacting the potential participants to further assess the couples’ relationship and their eligibility to enroll into the project.

### Validation of Dyadic Data Collected

For time- and resource-efficiency reasons, validation of couples’ data (ie, data came from two unique individuals) occurred after the algorithm electronically matched and verified that a couple was in a legitimate relationship. This approach helped to expedite the recruitment process by filtering out ineligible couples and couples whose relationships were not verified. Verified couples could then be validated. Validation of couples’ data was still required because some instances noted that the same IP addresses were being used in multiple screener entries. In some of these sets of entries with the same IP addresses, it was clear that the respondent(s) attempted the screener multiple times to determine the eligibility criteria to enroll in the study. Some individuals also created generic email addresses to appear as a couple for inclusion into the study. Because of these instances, the awareness of fraudulent yet eligible entries increased, thus warranting the creation of additional safeguards for a post hoc assessment of verified screener entries. Specifically, similar and/or suspicious email addresses (eg, “kylemcap1@gmail.com” and reporting partner’s to be “kylemcap2@gmail.com”) or names (eg, Melissa Wise) were noted and flagged. Also noted were similar yet different screener back-to-back entries to determine eligibility, same IP addresses used in multiple screener entries, and duplicate entries (ie, same two emails used for two different sets of couples). For these cases, the individuals and their “partners” were called and asked to verify, via phone, specific questions to assess their validity. Table 1 illustrates the difference between the manual and electronic methods of matching couples (verification of couples’ relationships) as well as examples of the post hoc assessment of screener entries (validation that data were from two unique individuals).

**Table 1:** Summary of the verification and validation of couples’ relationships and data for each of phase of the project.

Information used to verify and validate couples’ relationships	Developmental focus group phase	Pilot-testing randomized controlled trial phase
Number of screener items	17	17
Number of items used to determine eligibility	10	11
Predetermined items used to assess verification of relationship via comparison of self-reports by both partners of the couple	Individual’s birthday month; partner’s birthday month; individual’s age; partner’s age; relationship length; sexual agreement; individual’s email; partner’s email; individual’s phone number; partner’s phone number	Post hoc assessment
Method used to match and crosscheck predetermined items for relationship verification	Manual	Electronic via algorithm
Post hoc assessment of screener entries for validation purposes^a^	Similar/suspicious email addresses and names (eg, Kylemcap1@gmail.com and kylemcap2@gmail.com; Melissa Wise); back-to-back completions: time-stamped as 20-30 minutes apart (duration of survey); similarity of responses: consistent extreme/haphazard responses throughout survey; same IP addresses used in multiple entries: same IP address appearing in multiple screener entries with different responses (potentially to determine and pass eligibility criteria); duplicate entries: same two emails used for two different sets of couple IDs (eg, same two emails used for two different IDs: 2452EW and 2864OD)	

^a^ Experience suggested the continued need for post hoc assessment when using an electronic method and/or dealing with large sample sizes.

## Discussion

Several important lessons were learned about recruiting and collecting data from male couples from this project, resulting in recommendations for future Web-based studies specific to male couples. Because many MSM and/or male couples used the study Facebook webpage to ask questions or share stories/details, it was important to provide a friendly and supportive environment for these men to connect and communicate, free from public hostile comments.

For placement of targeted advertisements on Facebook, certain “interests” (eg, LGBT news) and images (ie, shirtless) should be avoided or used sparingly because they may generate adverse reactions from a broader audience. Although all images used in the advertisements were appropriate, some images may have been too explicit or hypersexualized for those outside the targeted audience, and especially to those who explicitly have a negative attitude toward the LGBT community. Researchers may also want to consider contacting established Facebook community groups (eg, Gay Miami Beach) who may have members of the targeted population to help promote the study. With respect to future work, researchers should consider how other characteristics of partnered MSM and male couples may influence the likelihood of them responding to targeted advertisements placed on Facebook. For instance, some images and recruitment text may resonate better with certain subgroups of male couples (eg, younger vs older male couples, ethnicity/race) and not others. Another consideration includes the target population’s computer and research literacy. As such, formative work is recommended to investigate which images, text, and placement of the advertisement (eg, desktop newsfeed, mobile newsfeed) would best help enhance the target populations’ response to clicking on the Facebook advertisement.

Experiences associated with screening potential male couples and verifying their relationships reinforces the need to develop and use a systematic approach that includes predetermined criteria with decision rules. These parameters will help enhance the efficiency as well as the reliability of collecting data from both members of the male couple. However, although time efficient, algorithms to electronically verify couples’ relationships may be imperfect and unable to capture certain nuances of their relationships. For example, two partners may define their relationship length differently if they had a break or period of separation occur during their relationship. Due to the discordance in their self-reports about relationship length, the predetermined decision rule associated with this questionnaire item would result in classifying this particular couples’ relationship as unverified. Based on the algorithm, this couple would then be deemed as unverified because of this one criterion they disagreed on. Researchers should consider which criteria they plan to use with corresponding decision rules because these decisions may affect the eligibility and verification of couples’ relationships in their Web-based projects that are specific to male couples.

It also is important to note that validation of dyadic data should not be assessed in isolation. Validation of dyadic data collected online should be based on examining multiple, if not all, predetermined criteria. Examining only one criterion (eg, same IP addresses, suspicious emails) may lead to disqualifying a potentially eligible verified couple. For instance, both partners of a male couple may live together and use the same computer, which would report them having the same IP address when completing a Web-based questionnaire. Email addresses that appear deceiving or misrepresentative of a potential participant warrant further investigation. For instance, an email address of “heather1@gmail.com” could be flagged as suspicious because the first name was Heather, a name typically associated with being a female. However, further investigation of this participant could reveal that his name was “Heath” and not “Heather.”

The Internet is a useful platform to target and enroll at-risk MSM and male couples from a variety of locales. However, it is evident that recruiting and collecting valid and reliable dyadic data online is more complex than that at the individual level. The varying results of the Facebook advertisement campaigns to recruit male couples further stresses the need to monitor posted Facebook advertisements to help minimize the receipt of negative feedback and that the target audience is being reached. Developing a systematic approach with predetermined criteria and parameters is strongly recommended to verify male couples’ relationships as efficiently as possible. Researchers are encouraged to develop a system of decision rules to detect and handle suspicious data to help validate the legitimacy that the dyadic data are coming from two unique partnered individuals. With the decision rules and criteria set, researchers should be cautious in observing multiple criteria as a whole, rather than in isolation. These lessons learned combined with recommendations for future studies may help other researchers enhance recruitment efforts and the validity and reliability of collecting dyadic data from male couples for novel preventive eHealth interventions specific to this population.

## Abbreviations

HIV: human immunodeficiency virus
IP: Internet Protocol
LGBT: lesbian, gay, bisexual, and transgender
MSM: men who have sex with men
RCT: randomized controlled trial
STI: sexually transmitted infection
