# Metadata Correction of: How Best to Obtain Valid, Verifiable Data Online From Male Couples? Lessons Learned From an eHealth HIV Prevention Intervention for HIV-Negative Male Couples

**DOI:** 10.2196/publichealth.6689

**Published:** 2016-09-26

**Authors:** Jason Mitchell, Ji-Young Lee, Rob Stephenson

**Affiliations:** ^1^ Office of Public Health Studies University of Hawai'i at Manoa Honolulu, HI United States; ^2^ University of Miami Miller School of Medicine Department of Public Health Sciences Miami Beach, FL United States; ^3^ Center for Sexuality and Health Disparities Department of Health Behavior and Biological Sciences University of Michigan School of Nursing Ann Arbor, MI United States

The academic affiliation of author Jason Mitchell was incorrect in the paper entitled “How Best to Obtain Valid, Verifiable Data Online From Male Couples? Lessons Learned From an eHealth HIV Prevention Intervention for HIV-Negative Male Couples” (JMIR Public Health Surveill 2016;2(2):e152). 

The affiliation of Jason Mitchell should be “Office of Public Health Studies, University of Hawai'i at Manoa, Honolulu, HI, United States”. The authors had brought this to the attention of JMIR editorial staff during proofreading; however, the error was only corrected in one of two instances where it needed to be edited.

 In addition, author Jason Mitchell has also changed the phone number from 1 808-956-8577 to 1 808-956-3342.

Both these alterations have been made in the online version of the paper on the JMIR website on October 3, 2016 together with publishing this correction notice. Because these were made after submission to PubMed and other full-text repositories, the correction notice has been submitted to PubMed, and the original paper has been resubmitted to PubMed Central. The corrected metadata have also been resubmitted to CrossRef.

